# Tuberculosis infection control knowledge and attitudes among health workers in Uganda: a cross-sectional study

**DOI:** 10.1186/s12879-016-1740-7

**Published:** 2016-08-15

**Authors:** Esther Buregyeya, Simon Kasasa, Ellen M. H. Mitchell

**Affiliations:** 1 Makerere University College of Health Sciences, School of Public Health, Kampala, Uganda; 2 KNCV Tuberculosis Foundation, The Hague, Netherlands

**Keywords:** Knowledge about TB infection control, Attitudes towards TB infection control, Health workers, Uganda

## Abstract

**Background:**

The World Health Organization recommends TB infection control (TBIC) in health care facilities. In 2008, the Ministry of Health Uganda initiated efforts to implement TBIC by training of health care workers (HCWs). This study was carried out to assess knowledge and attitudes towards TBIC among HCWs.

**Methods:**

We conducted a cross-sectional study among HCWs in health facilities in the districts of Mukono and Wakiso in Uganda, from October 2010 to February 2011. We assessed HCWs’ knowledge of basic standards of TB diagnosis, treatment and TBIC and attitudes towards TBIC measures.

**Results:**

Twenty four percent of the participants answered correctly all the basic TB knowledge questions. Overall, 62 % of the HCWs were judged to have adequate basic TB knowledge. At multivariable analysis, non-clinical cadres, were more likely to have poor basic TB knowledge, [adjusted odds ratio (aOR) 0.43; 95 % confidence interval (CI) 0.27–0.68)]. Only 7 % of the respondents answered all the questions on TBIC correctly. Almost all the respondents (98 %; 529/541) knew that TB was transmitted through droplet nuclei, while only a third (34 %; 174/532) knew that masks do not protect the wearer from getting TB. Overall, 69 % (355/512) of the HCWs were judged to have adequate TBIC knowledge. At multivariable analysis, non-clinical cadres (aOR 0.61; 95 % CI 0.38–0.98) and having not attended TBIC training, (aOR 0.65; 95 % CI 0.42–0.99), were more likely to have poor TBIC knowledge. More than three quarters (77 %; 410/530) and 63 % (329/522) of the respondents had a high self-efficacy and perceived threat of acquiring TB at work, respectively. Having not attended a TBIC training was significantly associated with a low self-efficacy (aOR 0.52; 95 % CI 0.33–0.81) and low perceived threat of acquiring TB infection at work, (aOR 0.54; 95 % CI 0.36–0.81).

**Conclusions:**

Our study finds moderate number of HCWs with correct knowledge and attitudes towards TBIC. Efforts should be put in place to train all HCWs in TBIC, with particular emphasis on the non-clinical staff due to their limited grasp of TBIC measures.

## Background

Transmission of tuberculosis (TB) to both patients and health care workers (HCWs) in health care settings has been reported from nearly every country, irrespective of local TB incidence [1, 2]. Transmission usually occurs from undiagnosed or inappropriately treated TB [3]. The risk for transmission varies by setting, occupational group, local prevalence of TB, patient population, and TB infection control (TBIC) measures in health care facilities [4–6]. TB has long been known as occupational hazard among HCWs [2, 4, 7]. Specific groups are at disproportionate risk including morgue technicians, housekeeping staff, laboratory workers [8]. The key factors facilitating nosocomial TB transmission include: delayed diagnosis, ineffective treatment of patients, and lack or inadequate TBIC measures [3, 9].

Uganda is among 22 countries with a high burden of TB. Multi-drug resistant TB (MDR-TB) accounts for 1.4 % among new patients and 12 % among retreatment [1, 10]. According to the WHO Global Report 2014, TB treatment success is 77 % [1]. Studies from Uganda found a high burden of TB among hospital staff, with a prevalence of 57 % for latent TB infection (LTBI), and 1.7 % compared to 0.3 % in the general population for active TB [11]. In addition, a high prevalence of LTBI was reported among medical students in Uganda [12]. The WHO recommends TBIC in health care institutions based on four levels: managerial, administrative, environmental and personal protective [3]. These measures have been found to minimize TB transmission [13, 14]. Therefore, it is recommended that all health facilities caring for TB patients or people presumed to have TB, implement TBIC [3]. In 2008, the Ministry of Health Uganda (MOH) and the Tuberculosis Assistance Programme (TBCAP) initiated efforts to implement TBIC by training of HCWs in selected districts, including Mukono district. In addition, the Uganda Ministry of Health produced TBIC guidelines [15] and rolled out the training in other districts in 2011.

Correct knowledge of a health problem, accompanied by the right attitude towards prevention, may result in healthy practices and behaviour [16, 17]. Previous research among HCWs in other countries has found that HCWs often lack knowledge about TB and infection control, which contributes to their increased risk [5]. This study was carried out to assess knowledge and attitudes towards TBIC among HCWs, in order to identify barriers to TBIC practices and to pinpoint specific groups who would benefit from mentoring and support on TBIC.

## Methods

### Study design, setting and population

We conducted a cross-sectional study among HCWs in health facilities in the districts of Mukono and Wakiso in central Uganda, from October 2010 to February 2011. These two districts surrounding Kampala, the capital city are semi-urban but predominantly rural. The HIV prevalence among the general population is estimated at 12.5 % among women and 8.4 % among men in Mukono and Wakiso [18]. In the two districts, training in TBIC was conducted 1–2 years preceding this survey. Health care in Uganda is provided by both public and private sector (private-not-for-profit - PNFP and private for profit). Uganda has a decentralised public health care system. At the lowest level is the Village Health Committee which acts as an outpost for outreach services at the village level, followed by health centre (HC) II at parish level (serving about 5000 people), HC III at a sub-county level (serving about 25,000 people), HC IV at the sub-district level (about 100,000 people) and the District Hospital. Each level offers services that the lower level provides in addition to services for its own level. TB services are offered at HC IIIs and above. For the purpose of this study, only public and PNFP health facilities from sub-county health facility (HC III) to hospital level (excluding those located on islands because of accessibility challenges) were included in the study. This is because TBIC training had only been conducted in the public and PNFPs health facilities. In Mukono district, the training was carried out by TBCAP, while in Wakiso district, the MOH Uganda did the training using the same training materials, with different facilitators and support. The objectives of the training in both districts were to teach HCWs a) how to conduct a TBIC assessment in a health facility and b) to develop and implement a TBIC plan in their facilities. Health facilities were asked to send to two people (usually a TB focal person and a laboratory technician) to attend the training. The trained individuals were supposed to transfer what they learnt to other HCWs through continuous medical education (CMEs) sessions in their respective health facilities. These CMEs are mainly geared towards (and attended by) HCWs who are directly involved in the management of patients.

### Sample size and sampling

A list of all health facilities within each district and the list of HCWs were obtained from the district health offices. Fifty-two health facilities were included in the study and the number of respondents from each health facility was proportional to the size of the facility in order to guarantee an equal probability of selection. HCWs were stratified by cadre in order to obtain a proportional representation of each staff category (doctors, clinical officers, nursing, midwifery, nursing aid, laboratory and radiographers). However, in facilities without all cadres represented, simple random sampling of all the HCWs in that facility was done. HCWs who were not present on the day of the study, for any reason were excluded from the study. Our calculated sample size was 551 HCWs. Full details of the methodology, including sample size calculation can be found in our previous publication [19].

### Data collection

A pre-tested self-administered questionnaire was used to collect data. We evaluated health workers’ knowledge of basic standards of TB diagnosis, and treatment as well as TBIC (knowledge about TBIC measures such as use of masks/respirators, triaging and ventilation) and attitudes towards TBIC measures. The questionnaire consisted of 20 questions in total, 7 for basic TB knowledge, 7 for knowledge on TBIC and 6 for attitudes towards TBIC. All the knowledge questions were true/false/ don’t know options (‘do not know’ answers were scored as incorrect), while those on attitudes were scored on five and three point likert scales, but were collapsed to two. The questions were adapted from the www.ghdonline tbic-baseline-assessment tool version 10 April 8 2009 with some modifications. One point was awarded for each correct answer. Thus the basic knowledge on basic TB and TBIC was allocated a minimum score of zero and a maximum of 7 each. Data were also collected on HCWs background characteristics including age, sex, qualifications, level of facility they were working in and type of facility in terms of ownership (public or private) and district of origin.

### Data management and analysis

Data were entered in Epi-Info Version 3.2.2 software and cleaned before being exported to STATA version 10 for analysis. Respondents were further categorized into clinical and non-clinical cadres. Clinical cadres included professionally qualified health providers (doctors/pharmacists, clinical officers, midwives, registered and certified nurses), while the rest were classified as non-clinical (nursing assistants and health management information system focal persons). Regarding basic knowledge on TB and TBIC, composite variables were created for each. These two variables were the outcome variables, while the explanatory ones were, age, sex, cadre, level of facility, district where the facility is located and ownership of the facility. For each outcome variable we computed a total score for every participant on the attributes, then the percentage by multiplying the total score by 100%. Respondents answering >85 % of the basic TB knowledge questions were considered to have good basic TB knowledge and others poor knowledge. Those answering >70 % of the TBIC knowledge questions were considered to have good TBIC knowledge and others poor knowledge. We used a lower cut off for TBIC knowledge (>70 %) compared to >85 % for basic TB knowledge, because TBIC is a new concept and not many of the HCWs may know it compared to basic TB knowledge.

Regarding attitudes towards TBIC, two separate composite variables were created; HCWs’ self-efficacy and HCWs’ perceived threat of acquiring LTBI at work. HCWs’ self-efficacy composed of the following three questions; i) There are things that I can do as a health worker to protect myself from TB, ii) 2. How much impact do you think you have to influence implementation of LTBI control measures in your facility; and iii) It is my responsibility to help identify TB suspects. “HCWs’ perceived threat of acquiring LTBI at work” composed of; i) My risk of contracting TB is NOT the same whether windows are open or closed; ii) There is need to screen health workers for TB; iii) How concerned are you about getting TB at your work. For each outcome variable we computed a total score for every participant on the attributes. These two variables were treated as ordinal variables with “0” meaning zero correct answer, “1” meaning one correct answer, “2” meaning two correct answers, and “3” meaning three correct answers. However, because of small numbers in the cells of “0” and “1” levels, respondents who answered zero to two correct answers were considered to have low self-efficacy/perceived threat, while those that answered correct all the 3 questions, as high self-efficacy/perceived threat. Bivariate analysis using Chi-square test and odds ratios (OR) were performed for basic TB knowledge, knowledge about TBIC, HCWs’ self-efficacy and HCWs’ perceived threat of acquiring LTBI at work. Logistic regression was used for multivariable analysis to explore the factors associated with all the four outcome. In addition, TBIC knowledge was used to predict self-efficacy and basic TB knowledge for perceived threat of acquiring B infection at work. Variables with *p* < 0.2 at bivariate level were included in the multivariable analysis model. An association was considered significant at *P* < 0.05.

## Results

### General characteristics

543 out of 551 HCWs (98.5 %) completed the questionnaire. The majority of the respondents (73 %) were females. The mean age of the respondents was 35.6 (10 standard deviation) years and median of 34 years [interquartile range (IQR) 27 to 43]. Over 80 % of the respondents belonged to the clinical cadre, as opposed to non-clinical. Among the clinical cadre, the majority were nurses (51 %), Table 1. The majority of the respondents worked in outpatient department (i.e., general outpatient department, maternal and child health services, pharmacy, HIV/ART clinic and laboratory). Less than half (45 %; 241/534) of the participants reported attending a TBIC training, with a significant difference by cadre (clinical and non-clinical; *p* = 0.002).

**Table 1:** Socio-demographic characteristics of the respondents in Mukono and Wakiso districts

Variable	n/N (%)
District	
Mukono	275/543 (51 %)
Wakiso	268/543 (49 %)
Facility level	
Hospital	253/543 (47 %)
HCIV	119/543 (22 %)
HC III	171/543 (31 %)
Facility ownership	
Government	351/543 (65 %)
PNFP	192/543 (35 %)
Sex	
Male	145/538 (27 %)
Female	393/538 (73 %)
Job category	
Doctor	18/540 (3 %)
Clinical officer	75/540 (14 %)
Registered nurse	83/540 (16 %)
Enrolled nurse	115/540 (21 %)
Midwife	75/540 (14 %)
Lab staff	69/540 (13 %)
Nursing assistants	81/540 (15 %)
Others	19/540 (4 %)
Age	
15–24	71/543 (13 %)
25–34	192/543 (35 %)
35–44	129/543 (24 %)
44 and above	151/543 (28 %)
Cadre	
Clinical	435/540 (81 %)
Non-clinical	105/540 (19 %)
Received training in TBIC	
Yes	241/534 (45)
No	293/534 (55)
Department	
Outpatient^a^	456/536 (85 %)
Medical ward	80/536 (15 %)

^a^Outpatient included the general outpatient department (OPD), HIV clinic, laboratory, pharmacy, records, and maternal child health services

Denominators vary for different variables because not all respondents answered all the questions

### Basic TB knowledge and determinants

The majority of the respondents (95 %; 515/541) knew that TB is the most common opportunistic infection affecting people living with HIV infection. Almost all (97 %; 527/541) of the respondents were aware that HIV increases the risk of developing TB disease, Table 2. The minimum knowledge score attained by the HCWs was 2 (28.5 %) of the expected maximum of 7 (100 %), with a median of 6 (85.7 %; IQR = 14.3). Twenty four percent (123/517) of the participants answered correctly all the questions about basic TB knowledge. Overall, 62 % (322/517) of the HCWs were judged to have adequate basic TB knowledge, based on the cut off >85.7 % (i.e., median score). At bivariate analysis, poor basic TB knowledge was significantly associated with female sex (OR 0.65; 95 % CI 0.43–1.00); not attending TBIC training (OR 0.58; 95 % CI 0.40–0.84) and being a non-clinical cadre (OR 0.37; 95 % CI 0.23–0.58). However, basic TB knowledge was not associated with age category, facility ownership, level of facility and where the HCW worked i.e., Wakiso or Mukono district and medical ward or outpatient, Table 3. At multivariable analysis, non-clinical cadres, were more likely to have poor basic TB knowledge, adjusted OR (aOR) 0.43; 95 % CI 0.27–0.68.

**Table 2:** Basic knowledge about tuberculosis and infection control among health workers in Mukono and Wakiso districts

Question		n/N	Percentage (%)
Basic TB knowledge			
TB is the most common opportunistic infection affecting PLWHA	Yes	515/5415	95
	No	26/54	5
HIV infection increases the risk of developing TB	Yes	527/541	97
	No	14/541	3
There is no difference between TB infection and TB disease	Yes	222/533	42
	No	311/533	58
A patient with suspected infectious TB should first be treated with broad-spectrum antibiotics before doing any investigations	Yes	192/540	36
	No	348/540	64
The first step in assessing a TB suspect is to send for a chest X-ray	Yes	104/537	19
	No	433/537	81
Sputum smear microscopy for AFB (Acid Fast Bacilli) is the quickest and cheapest way of identifying infectious TB patients	Yes	510/540	94
	No	30/540	6
All patients who have suspected infectious TB for the second time should have a sputum sent for culture and susceptibility testing	Yes	414/535	77
	No	121/535	23
TBIC knowledge			
How is TB transmitted?	Droplet	529/541	98
	Utensils and shaking hands	12/541	2
Covering the mouth when coughing has no effect on how many TB bacilli are expelled from an infectious TB Patient	Yes	154/535	29
	No	381/535	71
TB is more likely to be transmitted on TB wards as opposed to out-patient departments	Yes	236/538	44
	No	302/538	56
Is ventilation important in the implementation of TB infection control?	Yes	520/538	98
	No	18/538	2
A TB suspect should be placed in front of the queue in order to access services quickly	Yes	348/533	65
	No	85/533	35
Surgical masks do not protect the wearer against TB infection	Yes	174/532	34
	No	353/532	66
Sputum induction puts health workers at an increased risk of getting infected with TB	Yes	406/533	76
	No	127/533	24

**Table 3:** Bivariate and multivariable analysis of basic knowledge of TB among health care workers in Wakiso and Mukono districts in Uganda

Variable	Basic knowledge of TB		Univariate analysis	Multivariate analysis	P
	Good	Poor	Crude OR 95 % CI	Adjusted OR 95 % CI	
District					
Mukono	168/255 (66)	87/255 (34)	1	1	
Wakiso	154/262 (59)	108/262 (41)	0.73 (0.51–1.05)	0.83 (0.56–1.24)	0.37
Sex					
Male	96/138 (70)	42/138 (30)	1	1	
Female	226/376 (60)	150/376 (40)	0.65 (0.43–1.00)	0.72 (0.47–1.11)	0.14
Age					
15–24	39/67 (58)	28/67 (42)	1	-	
25–34	117/185 (63)	68/185 (37)	1.23 (0.69–2.18)	-	
35–44	77/124 (62)	47/124 (38)	1.17 (0.64–2.15)	-	
44 and above	89/141 (63)	52/141 (37)	1.22 (0.67–2.22)	-	
Facility level					
HCIII	108/167 (65)	59/167 (35)	1	-	-
HCIV	66/112/(59)	46/112 (41)	0.78 (0.47–1.28)		
Hospital	148/238 (62)	90/238 (38)	0.89 (0.59–1.35)	-	-
Facility ownership					
Government	208/337 (62)	129/337 (38)	1	-	-
PNFP	114/180 (63)	66/180 (37)	1.07 (0.73–1.55)	-	-
Cadre					
Clinical	278/416 (67)	138/416 (33)	1	1	
Non-clinical	43/100 (43)	57/100 (57)	0.37 (0.23–0.58)	0.43 (0.27–0.68)	<0.001
Attended TBIC training					
Yes	160/230 (70)	70/230 (30)	1	1	
No	160/280 (57)	120/280 (43)	0.58 (0.40–0.84)	0.70 (0.47–1.05)	0.08
Workplace					
In-patient	49/79 (62)	30/79 (38)	1	-	
Outpatient	272/434 (63)	162/434 (37)	1.02 (0.62–1.68)	-	

### Knowledge about TBIC and determinants

The minimum score on knowledge about TBIC attained by the respondents was 0 points (0 %) of the expected maximum 7 points (100 %), with a median of 5 points (71 %; interquartile range = 28.6). Only 7 % (35/512) of the respondents answered all the questions on TBIC correctly. Almost all the respondents (98 %; 529/541) knew that TB was transmitted through droplet nuclei, while a third (34 %; 174/532) knew that masks do not protect the wearer from getting TB, Table 2. Overall, 69 % (355/512) of the HCWs were judged to have adequate TBIC knowledge, based on the cut of >70 %. At bivariate analysis, poor TBIC knowledge was significantly associated with coming from Wakiso district (OR 0.64; 95 % CI 0.44–0.94); not attending TBIC training (OR 0.51; 95 % CI 0.34–0.76) and being non-clinical (OR 0.54; 95 % CI 0.34–0.86). However, knowledge about TBIC was not associated with age category, sex, facility ownership, level of facility and where the HCW worked i.e., medical ward or outpatient, Table 4. At multivariable analysis, being non-clinical (aOR 0.61; 95 % CI 0.38–0.97) and having not attended TBIC training (aOR 0.64; 95 % CI 0.42–0.99), were significantly associated with poor TBIC knowledge.

**Table 4:** Bivariate and multivariable analysis of TBIC knowledge among health care workers in Wakiso and Mukono districts in Uganda

Variable	TBIC knowledge		Univariate analysis	Multivariate analysis	P
	Good	Poor	Crude OR 95 % CI	Adjusted OR 95 % CI	
District					
Mukono	188/254 (74)	66/254 (26)	1	1	
Wakiso	167/258 (65)	91/258 (35)	0.64 (0.44–0.94)	0.71 (0.47–1.07)	0.10
Sex					
Male	103/137 (75)	34/137 (25)	1	1	
Female	252/372 (68)	120/372 (32)	0.69 (0.44–1.08)	0.77 (0.49–1.23)	0.28
Age					
15–24	40/63 (63)	23/63 (37)	1	-	
25–34	136/186 (73)	50/186 (27)	1.56 (0.85–2.87)	-	
35–44	87/123 (71)	36/123 (29)	1.39 (0.73–2.64)	-	
44 and above	92/140 (66)	48/140 (34)	1.10 (0.59–2.05)	-	
Facility level					
HCIII	112/166 (67)	54/166 (33)	1	-	
HCIV	75/112 (67)	37/112 (33)	0.98 (0.59–1.63)		
Hospital	168/234 (72)	66/234 (28)	1.22 (0.79–1.89)	-	
Facility ownership					
Government	228/332 (69)	104/332 (31)	1	-	-
PNFP	127/180 (71)	53/180 (29)	1.09 (0.74–1.62)	-	-
Cadre					
Clinical	297/411 (72)	114/411 (28)	1	1	
Non-clinical	58/99 (59)	41/99 (41)	0.54 (0.34–0.86)	0.61 (0.38–0.97)	0.04
Workplace					
In-patient	53/75 (71)	22/75 (29)	1	-	-
Outpatient	299/432 (69)	133/432 (31)	0.93 (0.54–1.59)		
Attended TBIC training					
Yes	174/225 (77)	51/225 (27)	1	1	
No	180/282 (64)	102/282 (36)	0.51 (0.34–0.76)	0.64 (0.42–0.99)	0.047

### Attitudes towards TB infection control measures

#### Self-efficacy

Almost all the respondents (97 %; 524/537) felt there are things they could do as HCWs to protect themselves from TB, Table 6. The majority (87 %, 464/536) reported that they had a moderate capacity to influence the implementation of TBIC in their facilities. More than three quarters (77 %; 410/530) of the respondents were considered to have a high self-efficacy. At bivariate analysis, being non-clinical cadre (OR 0.56; 95 % CI 0.34–0.91) and having not attended training in TBIC (OR 0.44; 95 % CI 0.28–0.69) were more likely to have a low self-efficacy, Table 5. At multivariable analysis, after controlling for cadre category and knowledge in TBIC, having not attended a TBIC training was significantly associated with a low self-efficacy (aOR 0.52; 95 % CI 0.33–0.81).

**Table 5:** Bivariate and multivariable analysis of HCWs self-efficacy towards TBIC in Wakiso and Mukono districts in Uganda

Variable	HCWs Self-efficacy towards TBIC		Univariate analysis	Multivariate analysis	P
	High, (n%)	Low (n,%)	Crude OR 95 % CI	Adjusted OR 95%CI	
District					
Mukono	209/266 (79)	57/266 (21)	1		
Wakiso	201/264 (76)	63/264 (24)	0.87 (0.58–1.31)	-	
Sex					
Male	108/142 (76)	34/142 (24)	1		
Female	299/383 (78)	84/383 (22)	1.12 (0.71–1.77)	-	
Age					
15–24	53/70 (76)	17/70 (24)	1		
25–34	153/192 (80)	39/192 (20)	1.25 (0.66–2.41)	-	
35–44	95/124 (77)	29/124 (23)	1.05 (0.53–2.08)		
44 and above	109/144 (76)	35/144 (24)	0.99 (0.51–1.94)		
Facility level					
HCIII	129/169 (76)	40/169 (24)	1		
HCIV	90/116 (78)	26/116 (22)	1.07 (0.61–1.88)	-	
Hospital	191/245 (78)	54/245 (22)	1.09 (0.69–1.75)		
Facility ownership					
Government	263/344 (76)	81/344 (24)	1		
PNFP	147/186 (79)	39/186 (21)	1.16 (0.75–1.78)	-	
Cadre					
Clinical	338/425 (80)	87/425 (20)	1	1	
Non-clinical	70/102 (69)	32/102 (31)	0.56 (0.34–0.91)	0.63 (0.38–1.04)	0.07
Workplace					
In-patient	60/78 (77)	18/78 (23)	1		
Out-patient	345/445 (78)	100/445 (22)	1.03 (0.58–1.83)	-	
Attended TBIC training					
Yes	197/232 (85)	35/232 (15)	1	1	
No	209/292 (72)	83/292 (23)	0.44 (0.28–0.69)	0.52 (0.33–0.81)	<0.01
TBIC knowledge					
Poor	111/155 (72)	44/155 (28)	1	1	
Good	276/347 (80)	71/347 (20)	1.54 (0.99–2.38)	1.27 (0.81–2.00)	0.29

#### Perceived threat of acquiring LTBI at work

Twenty one percent (112/537) of the HCWs mentioned that their risk of contracting TB was the same whether the consultation window was open or closed, Table 6. Almost two thirds (63 %; 329/522) of the respondents were considered to have a high perceived threat of acquiring LTBI at work. At bivariate level, a low perceived threat was significantly associated with being female (OR 0.62; 95 % CI 0.41–0.94); having not attended training in TBIC (OR 0.47; 95 % CI 0.33–0.69), and being non-clinician (OR 0.54; 95 % CI 0.35–0.84), Table 7. At multivariable analysis, not having attended TBIC training was significantly associated with having a low perceived threat of acquiring LTBI at work, (aOR 0.54; 95 % CI 0.36–0.81).

**Table 6:** Attitudes towards TB infection control measures among health care workers in Mukono and Wakiso districts

Variable		N	Percent
There are things that I can do as a health worker to protect myself from TB	Yes	524/537	98
	No	13/537	2
How much impact do you think you have to influence implementation of TB infection control measures in your facility	A lot	464/536	87
	Little/none	72/536	13
It is my responsibility to help identify TB suspects	Very much	474/537	88
	Somehow/Not at all	63/537	12
My risk of contracting TB is NOT the same whether windows are open or closed	Yes	425/537	79
	No	112/537	21
There is need to screen health workers for TB	Yes	447/537	83
	No	90/537	17
How concerned are you about getting TB at your work	Concerned	504/529	95
	Not concerned	25/529	5

**Table 7:** Bivariate and multivariable analysis of perceived threat of acquiring TB infection at work among HCWs in Wakiso and Mukono districts in Uganda

Variable	HCWs perceived threat		Univariate analysis	Multivariate analysis	P
	High, (n%)	Low (n,%)	Crude OR 95 % CI	Adjusted OR 95%CI	
District					
Mukono	171/261 (66)	90/261 (34))	1	-	
Wakiso	158/261 (61)	103/261 (39)	0.81 (0.57–1.15)		
Sex					
Male	100/141 (71)	41/141 (29)	1	1	
Female	226/376 (60)	150/376 (40)	0.62 (0.41–0.94)	0.68 (0.44–1.07)	0.09
Age					
15–24	42/68 (62)	26/68 (38)	1		
25–34	121/186 (65)	65/186 (35)	1.15 (0.65–2.05)	-	
35–44	77/122 (63)	45/122 (37)	1.06 (0.57–1.95)		
44 and above	89/146 (61)	57/146 (39)	0.96 (0.54–1.75)		
Facility level					
HCIII	108/168 (64)	60/168 (36)	1		
HCIV	71/116 (61)	45/116 (39)	0.87 (0.54–1.43)	-	
Hospital	150/238 (63)	88/238 (37)	0.95 (0.63–1.43)		
Facility ownership					
Government	209/344 (61)	135/344 (39)	1	1	
PNFP	120/178 (67)	58/178 (33)	1.34 (0.91–1.96)	1.28 (0.84–1.94)	0.25
Cadre					
Clinical	277/421 (66)	144/421 (34)	1	1	
Non-clinical	50/98 (51)	48/98 (49)	0.54 (0.35–0.84)	0.63 (0.39–1.02)	0.06
Workplace					
In-patient	50/75 (67)	25/75 (33)	1	-	
Out-patient	273/440 (62)	167/440 (38)	0.82 (0.49–1.37)		
Attended TBIC training					
Yes	168/230 (73)	62/230 (27)	1	1	
No	161/286 (56)	125/286 (44)	0.47 (0.33–0.69)	0.54 (0.36–0.81)	<0.01
Basic TB knowledge					
Poor	106/187 (57)	81/187 (43)	1	1	
Good	208/311 (67)	103/311 (33)	1.54 (1.06–2.24)	1.24 (0.84–1.86)	0.27

## Discussion

The study assessed HCWs knowledge and attitudes towards TBIC. Twenty four percent (123/517) of the participants answered correctly all the questions about basic TB knowledge. More than half (62 %) of the HCWs were judged to have adequate basic TB knowledge. Being non-clinical cadre was associated with having poor basic TB knowledge. Additionally, more than two thirds of the HCWs were judged to have adequate TBIC knowledge. Indeed, only 7 % of the respondents answered all the questions on knowledge about TBIC correctly. Knowledge about protection offered by the mask was the lowest with only 34 % of the respondents being aware that surgical masks do not protect the wearer against getting TB. Being a non-clinical cadre and not having attended TBIC training, were associated with having poor TBIC knowledge. Over half of the respondents had positive attitudes towards TBIC. Having not attended TBIC training was associated with a low self-efficacy and perceived threat to acquiring TB at work.

The findings that non-clinical staff and not attending TBIC training tend to have poor basic TB knowledge, TBIC knowledge and poor attitudes towards TBIC (low HCWs’ self-efficacy and perceived threat of acquiring LTBI at work), is similar to what was found in the Russian study where physicians and nurses were more knowledgeable than the support staff i.e., non-clinical [20]. This may be attributable to their different educational background and lack of priority for in-service IC trainings. Thus calls for the need to train non-clinical HCWs in TBIC in order to improve their knowledge and attitudes. This is because literature shows a heightened risk of getting TB disease among housekeeping staff [21]. The lower level of knowledge on use of masks is worrying. This is similar to what was found in a study done Ethiopia [22]. Knowledge of TBIC is similar to or higher than what was reported in the Ethiopian study, where 74.4 % of the respondents were found to have good TBIC knowledge [22], while in our study it is 69 %. This difference can be explained by a higher cut off of 70 % for our study, compared to the 60 % in the Ethiopian study.

Not having attended TBIC training was associated with poor TBIC knowledge and attitudes towards TBIC (HCWs’ self-efficacy and perceived threat of acquiring LTBI at work). This is consistent with findings from elsewhere that lack of trained staff is a big obstacle to TB control [22, 23]. This raises the importance of training all HCWs in TB and TBIC, i.e., institutionally based and in-service training/continuing education. Well educated HCWs with education needs tailored to job categories is critical for implementation of TBIC measures [3, 20]. A baseline assessment prior to the training can help identify both the strengths and weaknesses of the HCWs and thus enhance targeted training. Trainings for nursing aids and other lower cadres like cleaners and security guards can be specially arranged at the health facility to focus on their needs and gaps with regard to TBIC.

This study has limitations. We didn’t measure the practice of TBIC. Looking at TBIC practice would have given a complete picture. However, this was reported in one of the publications [24], where implementation of TBIC was found to be poor. The strengths of our study was to include all cadres of HCWs both clinical and non-clinical.

## Conclusions

More than half of the respondents were found to have good basic TB knowledge, TBIC knowledge and positive attitudes towards TBIC. Being a non-clinical cadre was associated with poor basic TB knowledge and TBIC knowledge. In addition, not having received training in TBIC was associated with poor TBIC knowledge and attitudes towards TBIC. These gaps in knowledge and attitudes recorded in this study can increase the risk of nosocomial transmission of TB. For effective implementation of TBIC, training in TBIC is critical with support staff (nursing aids, cleaners and security) being prioritized.

## Abbreviations

HCWs, health care workers; MDR-TB, multi drug resistant tuberculosis; SD, standard deviation; TBCAP, Tuberculosis Assistance Programme; TBIC, tuberculosis infection control
